# Mimicking the cell membrane: bio-inspired simultaneous functions with monovalent anion selectivity and antifouling properties of anion exchange membrane

**DOI:** 10.1038/srep37285

**Published:** 2016-11-17

**Authors:** Yan Zhao, Huimin Liu, Kaini Tang, Yali Jin, Jiefeng Pan, Bart Van der Bruggen, Jiangnan Shen, Congjie Gao

**Affiliations:** 1 Center for Membrane Separation and Water Science & Technology, Ocean College, Zhejiang University of Technology, Hangzhou 310014, P.R. China; 2 Department of Chemical Engineering, KU Leuven, Celestijnenlaan 200F, B-3001 Leuven, Belgium

## Abstract

A new bio-inspired method was applied in this study to simultaneously improve the monovalent anion selectivity and antifouling properties of anion exchange membranes (AEMs). Three-layer architecture was developed by deposition of polydopamine (PDA) and electro-deposition of N-O-sulfonic acid benzyl chitosan (NSBC). The innermost and outermost layers were PDA with different deposition time. The middle layer was prepared by NSBC. Fourier transform infrared spectroscopy and scanning electron microscopy confirmed that PDA and NSBC were successfully modified on the surfaces of AEMs. The contact angle of the membranes indicated an improved hydrophilicity of the modified membranes. A series of electrodialysis experiments in which Cl^−^/SO_4_^2−^ separation was studied, demonstrating the monovalent anion selectivity of the samples. The Cl^−^/SO_4_^2−^ permselectivity of the modified membranes can reach up to 2.20, higher than that of the commercial membrane (only 0.78) during 90 minutes in electrodialysis (ED). The increase value of the resistance of the membranes was also measured to evaluate the antifouling properties. Sodium dodecyl benzene sulfonate (SDBS) was used as the fouling material in the ED process and the membrane area resistance of modified membrane increase value of was only 0.08 Ωcm^2^ 30 minutes later.

The cell is the basic structure of the biosystem whose membrane has a multifunctional structure for the selective permeation of nutrient and ions in the cellular activity^1^,^2^. These fascinating multiple functions of the cell membrane are thought to be derived from the unique structure of its ionic and polar head groups of the phospholipid molecules and the fatty acid chains so that some small molecules can through it directly^1^,^3^,^4^. Therefore, the cell membrane structure has become a model for developing advanced membranes for ion separation^5^,^6^. From this point of view, inspiration from the cell membrane structure is undoubtedly a shortcut to the rational design for multifunctional anion exchange membranes (AEMs) in electrodialysis (ED)^7^.

ED is an effective electro-driven separation process to recover pure water from saline solutions^8^,^9^. Presently, ED process has been widely used in industrial applications such as industrial waste treatment processes, the desalination of seawater and separation of solutions containing organic ionic species^8^,^10^,^11^,^12^. For the lipid bilayer structure of cellular membranes, it can be seen that as the polar head groups that distribute in the innermost and outermost of the structure and the fatty acid chains inside the structure. Inspired by this special “three-layer” structure, it may be significant to develop and research new membranes in order to improve the monovalent anion selectivity and antifouling property.

With the development of science and technology, the separation of monovalent and divalent anions in aqueous solution is important for industrial applications^13^. Traditionally, designing a layer with proper negative charge and dense architecture on the surface of AEMs appears to be an effective way to improve the monovalent anions’ selectivity. Such modifications could involve a cross-linked monovalent anion permselectivity layer or a layer-by-layer modification and these methods have achieved excellent results of the monovalent anions selectivity^14^,^15^,^16^. However, the existence of the membrane fouling on the membrane surface may hinder the monovalent selectivity. Generally, the membrane fouling is caused by complex physical and chemical interactions which is a frequent drawback for all membrane processes^17^. During water treatment, the fouling of mineral scaling, organic fouling and biofouling especially need to be considered^18^,^19^,^20^. Organic fouling, caused by protein and surfactant adsorption or aggregation on hydrophobic surfaces, is a very common problem during water treatment^21^,^22^,^23^,^24^. In general, water is used to clean the fouled membrane surface. However, it is difficult to fully remove the organic materials of AEMs^25^. A current strategy for controlling membrane fouling is to cross-link an antifouling layer on the surface of the AEMs^26^,^27^,^28^,^29^. To this end, exploration of the structure of the membrane may have a new breakthrough.

Bio-inspired artificial single ion channels from the aquaporin of cell membranes and control ionic transport which have successfully mimicked the cooperative response double gates of biological ion pumps^30^,^31^. Unfortunately, membranes with monovalent anion selectivity and antifouling properties are seldom reported. Thus, inspiring from the special “three-layer” structure of cell membrane to simultaneously improve monovalent anion selectivity and antifouling properties may break through this problem. Dopamine as an antifouling material was self-assembled on the surface of membranes to form a bioadhesive layer with microporous architecture^32^,^33^,^34^. Polydopamine (PDA) layer was formed on the surface of AEMs by deposition of dopamine with self-polymerization in the presence of oxygen^34^. The mechanism and the chemical mechanism functions of membranes with the PDA layer are also improved^35^,^36^,^37^. N-O-sulfonic acid benzyl chitosan (NSBC) was synthesized by a Schiffbase formation reaction^38^ and the main raw material of NSBC is the deacetylated product of chitosan, which is a natural polymer^39^. NSBC can be used to improve the monovalent anion selectivity due to its –SO_3_^−^ groups.

Thus, inspired by the special “three-layer” structure of cell membrane, we develop a modification of “three-layer” structure on the surface of AEM. Ions of Cl^−^ and SO_4_^2−^ were used to determine the permselectivity and the antifouling properties were measured by sodium dodecyl benzene sulfonate (SDBS) to preliminary explore the monovalent anion selectivity and antifouling properties of modified AEM, simultaneously.

## Results and Discussion

### Surface characterization of the membranes

**Figure 1:**
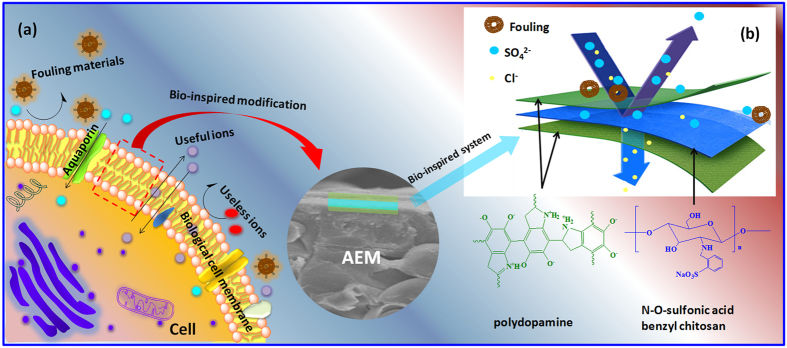
Schematic of the bio-inspired “three-layer”modification, bio-inspired modification of AEMs (**a**) and the monovalent anion selectivity and antifouling property of each layer (**b**).

The bio-inspired modification of AEMs was shown in Fig. 1(a). The monovalent anion selectivity and antifouling property of each layer was shown in Fig. 1(b).

**Figure 2:**
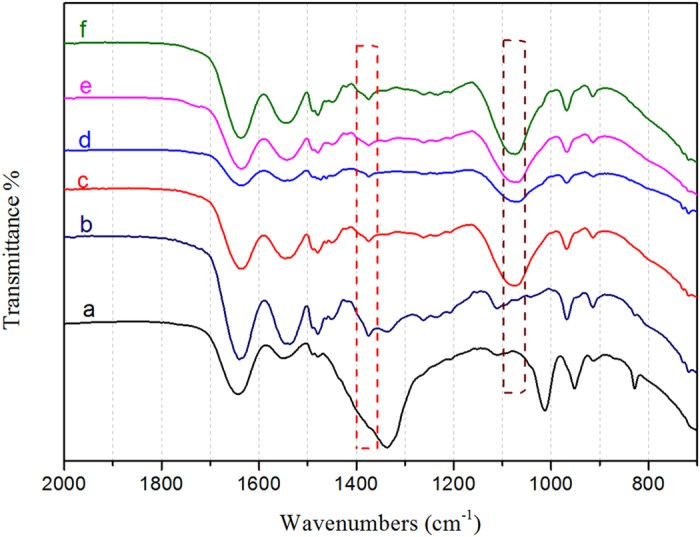
FT-IR spectra of unmodified membrane (**a**) DPA modified membrane without NSBC (**b**) and the modified membranes, membrane 1 (**c**) membrane 2 (**d**) membrane 3 (**e**) membrane 4 (**f**).

In Fig. 2, FTIR spectra were used to confirm the chemical architecture of the membrane surface. The absorption bands at 1375 cm^−1^ are the characteristic bands of –OH (primary hydroxyl). All the modified membrane samples as seen in Fig. 2(b), (c), (d), (e) and (f) show this characteristic peak except for the unmodified AEM. This is because the PDA and NSBC contain primary hydroxyl groups, but the unmodified AEM does not. Furthermore, the spectrum of “three-layer” modified membrane as seen in Fig. 2(c), (d), (e) and (f) shows strong sulfonate absorbance at 1075 cm^−1^ and 1030 cm^−1^ vibration modes. The adsorption bands are due to symmetric stretching vibrations of -SO_3_H and symmetric stretching vibrations of S=O. These spectra are different from the modified membrane, which was only coated in PDA layer as shown in Fig. 2 spectra (b).

**Figure 3:**
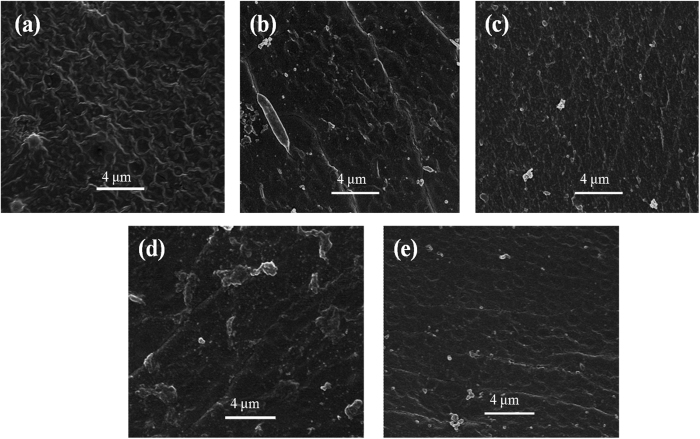
SEM images of AEMs: unmodified membrane (**a**) modified membrane 1 (**b**) modified membrane 2 (**c**) modified membrane 3 (**d**) and modified membrane 4 (**e**).

**Figure 4:**
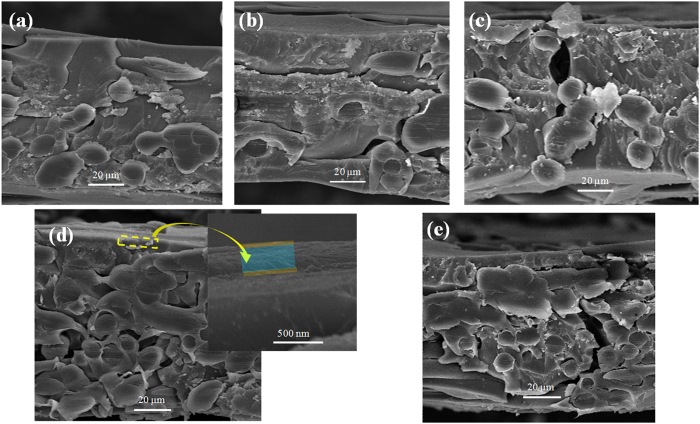
Cross-section SEM images of unmodified AEM (**a**) and modified membrane 1 (**b**) modified membrane 2 (**c**), modified membrane 3 (**d**) and modified membrane 4 (**e**). In order to observe the bio-inspired architecture, the modified membrane 3 (**d**) modified layer was amplified specifically.

In order to further characterize the modified membrane, the surface and cross-section of the AEMs were observed by SEM. Figure 3 shows the surface images of AEMs. The unmodified membrane shown in Fig. 3(a) has a rugged shape surface. This kind of commercial AEM has the poor antifouling properties we previously described. Figure 3(b) and (c) are modified membrane 1 and modified membrane 2 respectively, and the surface of modified membrane 2 is smoother because of the different modification architecture of the NSBC layer. However, the surface of modified membrane 3 as shown in Fig. 3(d) with the middle electro-deposition NSBC with 15 mA/cm^2^ has no crater-shape structures. In Fig. 3(e), the surface is different from others because of the decrease of the deposition time of innermost PDA layer. Correspondingly, Fig. 4(a), (b), (c), (d) and (e) are the cross-sectional images of the AEMs. The architecture of the AEM composed of the membrane matrix and the bio-inspired architecture can be observed in these micrographs. From Fig. 4(a), the unmodified AEM shows no layer while the modification layers can be distinguished clearly,as shown in Fig. 4(b), (c), (d) and (e). In order to observe the “three-layer” architecture, this bio-inspired section was amplified specifically. The thickness of the modified three layers of modified membrane 3 is about 200 nm.

**Figure 5:**
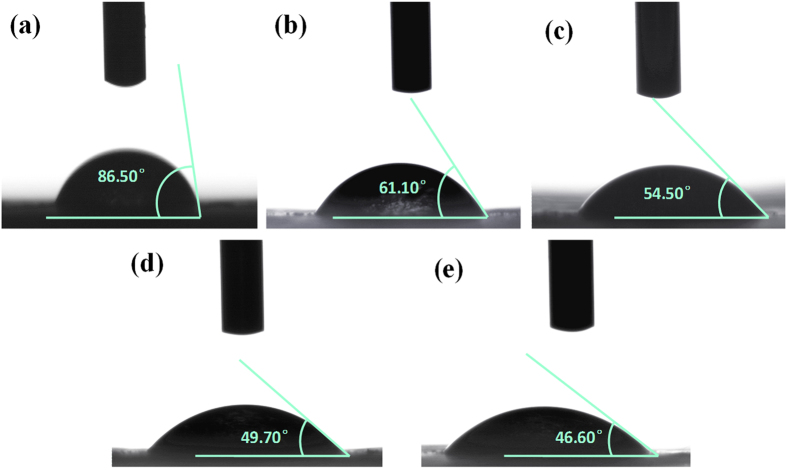
Contact angle at 5 seconds of unmodified membrane (**a**) modified membrane 1 (**b**) modified membrane 2 (**c**) modified membrane 3 (**d**) and modified membrane 4 (**e**).

The contact angle measurements of AEMs are shown in Fig. 5. The water contact angle of the unmodified AEM surface is 86.5° (Fig. 5(a)). When the membrane is modified, the water contact angle decease, which means the hydrophilic properties have been augmented. The effect of the bio-inspired layer was to cause the increase of hydrophilic properties with lager number of hydrophilic groups in PDA layers and NSBC layers. This phenomenon may have led to the improvement the antifouling properties of AEM.

As shown in Table 1, the unmodified membrane is a commercial AEM and the membrane area resistance is 1.30 Ωcm^2^. After modification, the modified membrane 1 area resistance is 1.37 Ωcm^2^, which is only a very small increase. However, the modified membrane 2 area resistance is 1.81 Ωcm^2^ and that of the modified membrane 3 is 1.94 Ωcm^2^. Thus, it is shown that the area resistance increases with the thickness of the NSBC layer. When the keep same thickness of the NSBC layer as for membrane 3, but the innermost self-deposition of PDA at 2 hours (modified membrane 4), the area resistance is 1.87 Ωcm^2^.

### Electrochemistry

In recent years much effort has been devoted to developing a new kind of AEM to improve the monovalent anion selectivity or antifouling property. There are many methods and theoretical studies on treating the effect of parameters on the permselectivity of anions and membrane fouling problems. Most organic foulants have the negative charge which gives the research direction of antifouling properties of AEMs^22^ and many attempt to increase the function of antifouling of AEMs have reported^17^,^40^,^41^,^42^. There are many reports that PDA coating can enhance the anti-organic fouling performance of a commercial AEM by imparting high hydrophilicity and negative charge on the AEM surface^43^,^44^,^45^. Theoretically, PDA on the surface of AEMs has the monovalent anion permselectivity potential simultaneous and a report has shown that theoretically, which has given the proof^46^.

In this study, we attempted to simultaneously improve the antifouling property and the monovalent anion selectivity of AEM by inspiring the special “three-layer” structure modified membrane and the main materials were the PDA and NSBC. Here, we used the electrochemical characterization of the modified membranes (polarization current-voltage curves of AEMs modified by PDA layer and “three-layer” structure) to show the effect of PDA and NSBC layer. Polarization current-voltage curves were measured in a four-electrode mode under direct current to characterize the electrochemical behavior of the modified membranes (see Figure S1 in Supporting Information).

Current-voltage curves are important to characterize the electrochemical behavior of the modified membranes. The current-voltage curves show the different behaviors of AEMs over a wide range of currents and display three different regions (Ohmic region, plateau region and over limiting region). The first region is Ohmic region and happened at low current densities. In this region, the potential drop across the membrane is directly proportional to the applied current. The second region is called “plateau” region which happens when the current density increases and approaches its diffusion-limited value (*I*_*lim*_), ions transport through the membrane will be more quickly. Thus, the concentration of anions in the dilute side near the AEM will rapidly cause an increase in resistance and form smaller slope. The third is the over limiting region, where the current density increased to a threshold. This will form an advanced stage of concentration polarization in the solution and that may destabilize the boundary layer. In this region, the current density values increase again while the membrane voltage drops.

**Figure 6:**
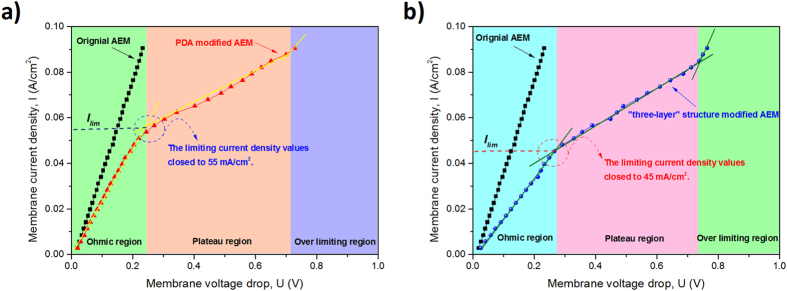
Current-votage curves of unmodified and PDA modified AEMs (**a**); unmodified and membrane 3 (**b**).

Figure 6(a) and (b) show the current density-voltage curves for the unmodified AEM, PDA modified membrane and “three-layer” structure modified AEM (in this work, we choose the modified membrane 3 as the example). With regard to the limiting current density, as can be seen from Fig. 6(a), unmodified membrane exhibits only an Ohmic profile under the same conditions. Because its limiting current is much higher than the modified membrane due to the absence of the restrictive membrane and more importantly passage of both SO_4_^2−^ and Cl^−^. When this kind of membrane is modified only by PDA, three different regions can be obviously distinguished and the limiting current density values is close to the 55 mA/cm^2^. In Fig. 6(b), one can see the current density-voltage curve of unmodified AEM and modified membrane 3. The three different regions of modified membrane 3 also significantly appeared in the current density-voltage curve. Because SO_4_^2−^ will not significantly through the modified membrane and a negligible effect on I_lim_ for the modified membrane, it will not affect I_lim_ for the unmodified AEM. However, when we compare Fig. 6(a) and (b), the limiting current density values closed to the 55 mA/cm^2^ of PDA modified AEM and the limiting current density values closed to the 45 mA/cm^2^ of “three-layer” structure modified AEM. The divergence of the limiting current density values may illustrate that the “three-layer” structure modified AEM has greater impact on I_lim_ than PDA modified AEM. This result can show that the “three-layer” structure modified AEM has more excellent monovalent anions selectivity than PDA modified membrane. Specifically, the result of the current density-voltage curve can show the importance of NSBC in monovalent anions selectivity.

### Evaluation of monovalent anion permselectivity on Cl^−^/SO_4_^2−^ separation

**Figure 7:**
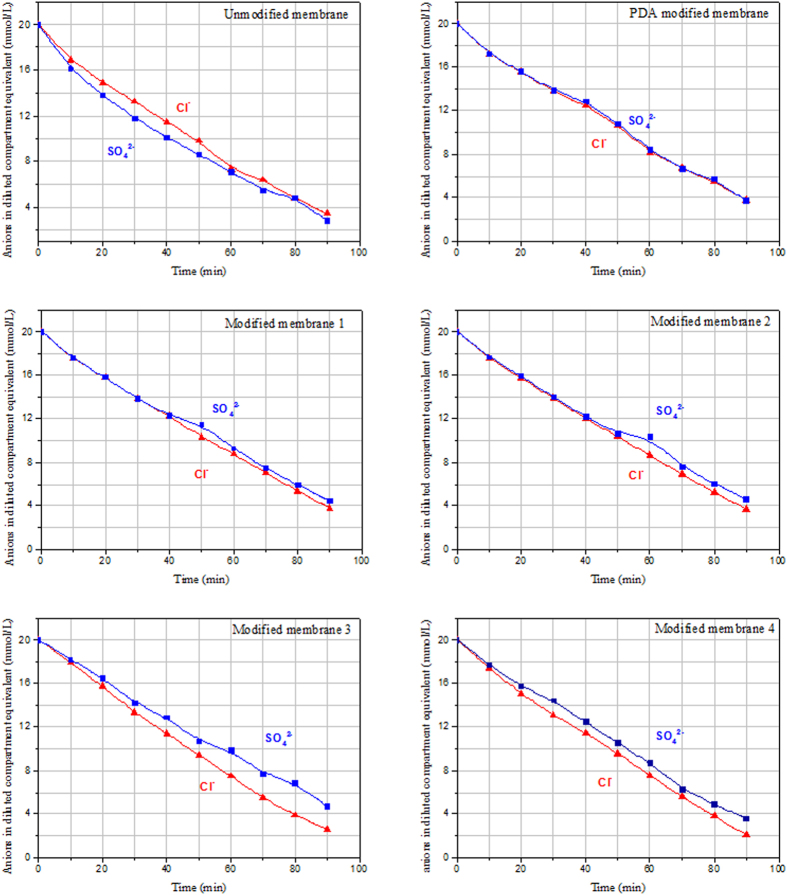
Temporal evolution of Cl^−^ and SO_4_^2−^ ions transport of the unmodified AEM, modified AEM without NSBC layer and “three-layer” modified AEM.

The monovalent anion permselectivity of the modified AEMs was evaluated by measuring the relative concentrations of Cl^−^ and SO_4_^2−^ ions in the dilute compartment. Temporal evolution of anions transport is shown in Fig. 7. For the unmodified membrane, the concentrations of Cl^−^ and SO_4_^2−^ ions in the dilute compartment decrease at the same time in ED process and the concentrate of Cl^−^ always keep higher than SO_4_^2−^ ions in the dilute compartment. The phenomenon can be explained by the transport of anions under the DC constant current and the migration rate depending on the value of anions charged particles. This phenomenon reflects that this unmodified AEM has poor monovalent anions selectivity. When the AEM is modified by PDA without NSBC, the Cl^−^ ions in the dilute compartment lower than SO_4_^2−^ which show the opposite phenomenon than the unmodified AEM, but this phenomenon cannot be distinguished clearly. As shown in Fig. 7, When the AEMs surface with the “three-layer” modification structure, the concentration of Cl^−^ ions in the dilute compartment lower than SO_4_^2−^ and these phenomena can be clearly distinguished. In addition, Cl^−^ ions in the dilute compartment decrease faster than that of SO_4_^2−^ and the decreases rates of these four kind of modified membranes were also different. The modified membranes not only show the monovalent anions permeate easier through the membrane than multivalent anions but also show the significance of NSBC layer in the “three-layer” bio-inspired modification structure.

**Figure 8:**
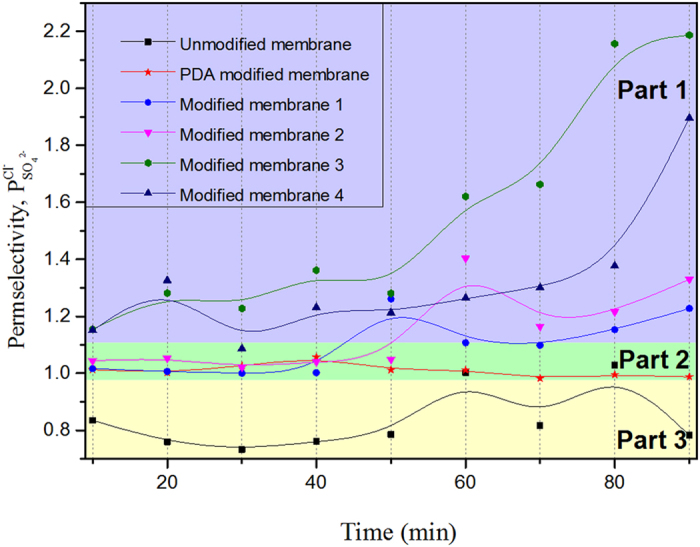
The permselectivity of Cl^−^/SO_4_^2−^ of the modified and unmodified AEM.

The permselectivity values were obtained by measuring the concentration change of Cl^−^ and SO_4_^2−^ ions in the dilute compartment, shown in equation (1) and Fig. 8, the permselectivity of Cl^−^/SO_4_^2−^ of the modified and unmodified AEM. The key factor of the monovalent anion permselectivity was the difference of ionic mobility in the membrane. In Fig. 8, three parts represent the membranes with obvious permselectivity, inconspicuous permselectivity and no permselectivity. In Fig. 8 part 3, we can observe that the permselectivity of unmodified membrane was lower than 1 which revealed that the unmodified membrane has no monovalent anions permselectivity. In part 2, the AEM modified by PDA without NSBC and the permselective is higher than 1, but it is very close to 1 which means that this kind of modified membrane has the inconspicuously monovalent anions permselectivity. In part 1, the permselectivity of modified membranes is higher than the PDA modified membrane. This can be explained that for the modified AEM, the migration of monovalent anions is higher than that of the divalent anions. The improved monovalent anion permselectivity of modified AEM improved can be ascribed to the NSBC layer, which contains the negatively charged ions in the three-layer architecture. Compare to the unmodified membrane whose permselectivity is 0.78 in 90 min, but the permselectivity of modified membrane (especially modified membrane 3) even reach at 2.20. Hence, the monovalent anion selectivity was due to the bio-inspired modification.

### Evaluation of the antifouling property

**Figure 9:**
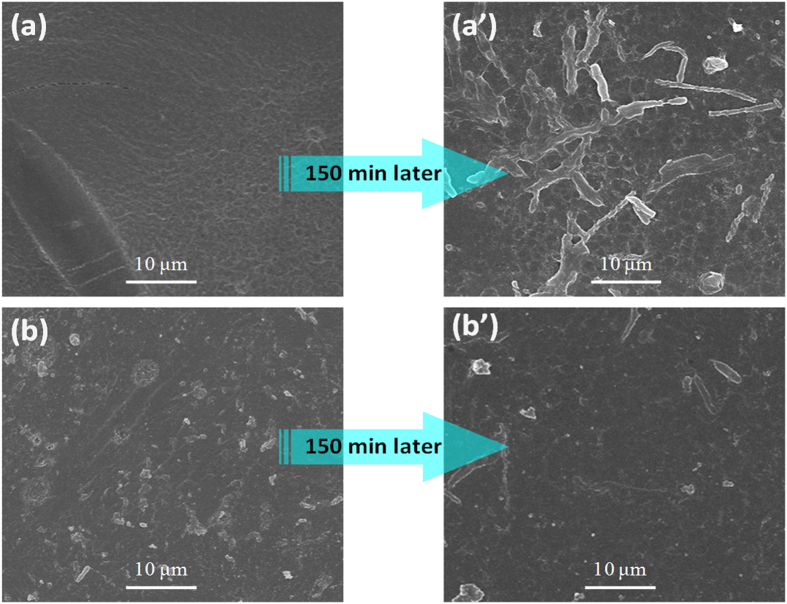
SEM images of the membranes surface: the unmodified membrane (**a**) and modified membrane (**b**). After 150 min later, the unmodified membrane surface fouling by SDBS (**a**’) and the modified membrane fouling by SDBS (**b**’).

In the previous section, it was demonstrated that the hydrophilicity of the modified membranes had been improved, which affected the antifouling potential of AEMs. Therefore, it was expected that the modification of AEMs may have a high antifouling potential. Sodium dodecyl benzene sulfonate was used as the membrane fouling material in the ED process. The modified membrane 3 was selected to evaluate the antifouling potential. The membrane area resistance is shown in Table 2. The increase value of the area resistance of the modified membrane after 30 minutes was 0.08 Ωcm^2^, while the increase value for the unmodified membrane increases is 0.6 Ωcm^2^. After 90 min, the area resistance was further increased for both the unmodified membrane and the modified membrane, but the increase value of the modified membrane was only half of the increase value for the unmodified membrane in this interval. After 150 min, the area resistance seems to reach a plateau value. SEM images (shown in Fig. 9) of the membranes surface were used to characterize the change of the membrane surface. Figure 9(a) and (b) show the shows smooth and clean surface at beginning of commercial unmodified and modified membrane surface respectively. While after 150 minutes, the fouled surface can be seen in Fig. 9(a’). The commercial unmodified AEM were fouled by SDBS heavily during the ED process. However, for the modified membrane, the surface was much smoother and cleaner after the same procedure, as shown in Fig. 9(b’). Comparing Fig. 9(a’, it can be concluded that the AEM antifouling property has been improved by bio-inspired modification.

## Methods

### Chemicals and membranes

**Figure 10:**
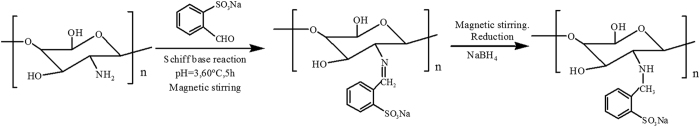
Reaction route for NSBC.

For this study, dopamine hydrochloride (HWRK Chem. Co., Ltd., Beijing, China) was used to modify the AEM. Tris-buffer (Energy Chemical Co., Shanghai, China), sodium borohydride (Guanghua Chemical Factory Co., Ltd., Guangdong, China), sodium dodecyl benzene sulfonate (Yingpeng Chemical Co., Shanghai, China) were used to synthesize NSBC. NSBC was synthesized according to the Schiffbase formation reaction (see Figure S2 in Supporting Information); the reaction route for the NSBC is shown in Fig. 10. All other chemicals were obtained from Aladdin Industrial Co. (Shanghai, China).

In this study, the commercial Homogeneous AEMs (FUJIFILM, Japan) were used to be modified. The commercial Homogeneous cation exchange membranes (CEMs) (FUJIFILM, Japan) were used to prevent the leakage of anions in the lab-made apparatus. The properties of the commercial AEMs and CEMs are shown in Table 3.

### Procedure for membrane modification

First, dopamine was selected to coat the commercial AEM surface and formed a PDA layer in the innermost of the modification structure. Second, under the electro-deposition methods, NSBC was chosen to form the middle layer. Lastly, the outermost layer of the modification structure was also the PDA layer.

**Figure 11:**
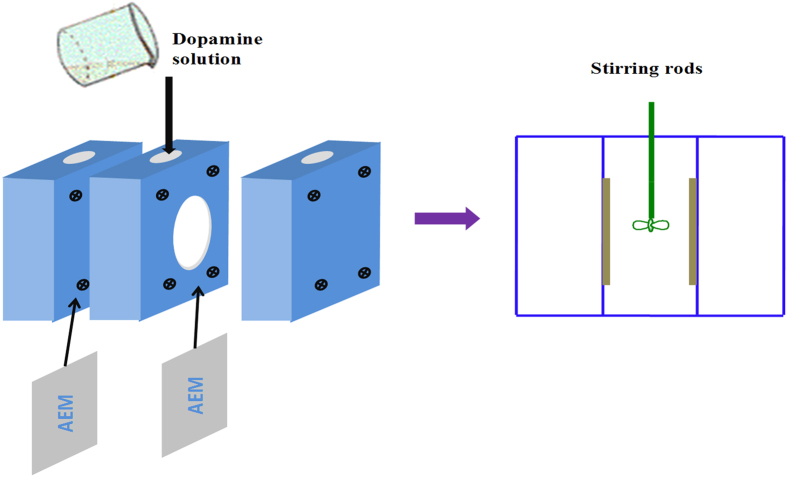
Experimental setup for deposition of the dopamine on the surface of the membrane to form a PDA layer.

**Figure 12:**
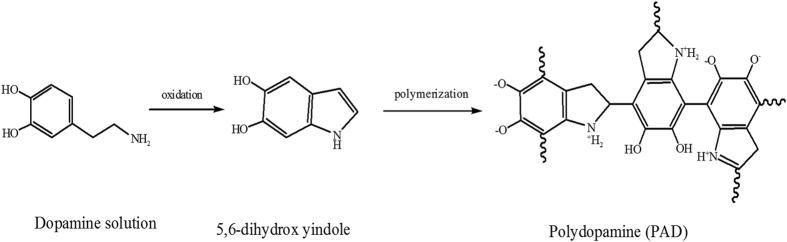
Reaction route for the PDA.

The innermost PDA layer was prepared by dissolving 0.20 g of dopamine into 100 mL of a 15 mM Tris-HCl buffer (pH 8.8). Then the solution was added into the lab-made experimental setup as shown in Fig. 11. The stirring rods were used at a speed of 100 rpm. The color of the solution changed from clear to darkish brown when in contact with oxygen. After stirring for 3 hours (membrane 4 was 2 hours), particulate impurities on the surface of PDA were rinsed off by pure water. Dopamine self-polymerizes easily to form a thin layer on the surface of AEMs; the reaction route for the PDA is shown in Fig. 12.

**Figure 13:**
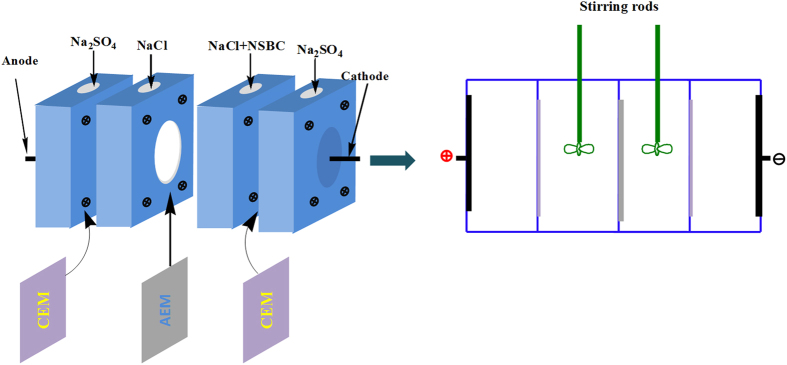
Experimental setup for electrodeposition of the NSBC layer (the anode was the ruthenium coated titanium electrode and the cathode was the stainless steel electrode).

The second layer (middle layer) was the NSBC and the solution was 1 g/L (with 0.5 M NaCl as a supporting electrolyte). As shown in Fig. 13, the AEM was clamped in the middle of cells and two auxiliary CEM membranes were used. The experimental setup for electro-deposition of the NSBC layer was at current densities of 5 mA/cm^2^, 10 mA/cm^2^ and 15 mA/cm^2^, respectively. After the above process, the membrane was rinsed in pure water. The outermost layer was formed by deposition of dopamine in 2 hours with the same step of innermost PDA layer. In between the coating steps, the membranes were thoroughly rinsed in pure water. Lastly, the modified membranes were immersed into pure water.

In this paper, different factors modifications of AEMs were marked in the different names, as shown in Table 4.

### Surface characterization of the membranes

The changes of the functional groups on the commercial AEM and modified AEMs were characterized by Fourier Transform infrared spectroscopy (ATR-FTIR) (Nicolet 6700) at room temperature to provide information about the chemical structure of the commercial and modified membranes. The membrane surface and cross-sectional structures of the membranes were examined using scanning electron microscopy (SEM) (S-4700 Hitachi, Tokyo, Japan) and water contact angles of the supports and membranes were measured using the sessile drop method on a goniometer.

### Permselectivity measurements and calculation

The AEMs permselectivity was measured under the application of a current density of 5 mA/cm^2^. Permselectivities were measured in a four compartment module for monovalent anion permselectivity measurement (see the drawing of a four-electrode mode see Figure S3 in Supporting Information). In the middle of the two compartments filled with the same salt solution, i.e., 0.02 M equimolar mixtures of sodium salts of mono- and divalent anions (Cl^−^ and SO_4_^2−^). In the dilute compartment, the concentration of mixtures Cl^−^ and SO_4_^2−^ was measured by Anion Chromatography every 10 minutes (total duration 90 minutes).

The permselectivity of the membranes between chloride and sulfate ions (Cl^−^ and SO_4_^2^) 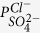 was calculated as the eq. (1).


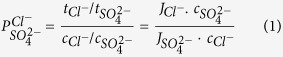


where *t*_*i*_ is the transport number of the ions through the membrane, *Ji* is the flux of the ions through the membrane expressed in mol/m^2^·s and *c* is the concentration of the ions in the diluate compartment, expressed in mol/L. The *t*_*i*_ was calculated using eq. (2).


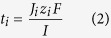


where *z*_*i*_ is the ion charge, *F* is Faraday’s constant and *I* is the DC current expressed in A cm^−2^. The flux of ions was obtained from the change in concentration of the ions on the dilute side according to eq. (3).


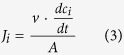


where *v* is the volume of the electrolyte solution in dilute compartment which is 1 L, and *A* is the active area of the membranes, which was 19.625 cm^2^.

### Antifouling measurements and calculation

The increase value of the modified and unmodified membrane area resistance was measured every 30 minutes. SDBS was used as the membrane fouling material in the four-compartment experimental setup (as shown in Figure S5 in Supporting Information). In this study, 0.2 g/L NSBC was used as the fouling materials and mixtures of 0.1 M NaCl solution in one of the middle compartments, the other one was the 0.1 M NaCl solution. The two sides of compartments were the electrode chamber and the electrode solution was a solution of 0.2 M Na_2_SO_4_. With the DC constant current in 5 mA/cm^2^, the antifouling properties of the commercial AEM and modified membrane 3 were studied. The fouling process was evaluated after 0 min, 30 min, 90 min, 150 min, and 210 min. Membrane area resistance measurements were carried out separately in 0.5 M NaCl solution at room temperature, using a custom-designed cell (see Figure S4 in Supporting Information).

It was measured via the setup and calculated via the equation


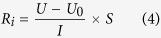


where *R*_*i*_ is the resistance of the unmodified and modified membrane area reactance expressed in Ω·cm^2^, *U* is the voltage of the membrane and *U*_*0*_ is the voltage of blank expressed in V, *I* is the constant current through the membrane and insure the current at 0.004 A, *S* is the membrane effective area in this setup (7.065 cm^2^).

Compared to the beginning of the membrane area reactance, we found the increase in the membrane area resistance was calculated according to


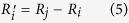


where 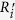 is the increase value of the membrane area resistance and *R*_*j*_ is the membrane area resistance of modified and unmodified area resistance of AEM which was fouling during ED process after several minutes later, expressed in Ω·cm^2^.

## Summary

The commercial AEM was modified by PDA and NSBC, which was the “three-layer” bio-inspired modification structure on the surface of the AEM. This structure, inspired by the special “three-layer” structure of cell membrane effectively improves the monovalent anion selectivity and antifouling properties. The results of Cl^−^/SO_4_^2−^ permselectivity show that the migration of monovalent anions of modified AEMs was higher than the divalent anions. The permselectivity of anions reached up to 2.20 in 90 min later. SDBS was used as fouling material in ED process to evaluate the antifouling property. The membrane area resistance of the unmodified membrane was increased by more than 0.63 Ωcm^2^ in 30 min while the membrane area resistance increase value of modified membrane was only 0.08 Ωcm^2^. Besides, the membrane resistance increase value of the modified membrane was half of the commercial membrane after 150 minutes.

## Additional Information

**How to cite this article**: Zhao, Y. *et al.* Mimicking the cell membrane: bio-inspired simultaneous functions with monovalent anion selectivity and antifouling properties of anion exchange membrane. *Sci. Rep.* **6**, 37285; doi: 10.1038/srep37285 (2016).

**Publisher’s note:** Springer Nature remains neutral with regard to jurisdictional claims in published maps and institutional affiliations.

## Supplementary Material

Supplementary Information

## Figures and Tables

**Table 1:** Membrane area resistances and the absolute increase value of membrane area resistance of unmodified membrane and modified membranes.

Membrane type	Area resistance (Ωcm^2^)	Absolute increase value of membrane area resistance (Ωcm^2^)
Unmodified membrane	1.30	0
Modified membrane 1	1.37	0.07
Modified membrane 2	1.81	0.51
Modified membrane 3	1.94	0.64
Modified membrane 4	1.87	0.57

**Table 2:** Temporal evolution of the unmodified and modified AEMs area resistance increase value.

Time	Commercial AEM area resistance (Ωcm^2^)	Absolute increase value (Ωcm^2^)	Modification AEM area resistance (Ωcm^2^)	Absolute increase value (Ωcm^2^)
0 min	1.30	0	1.94	0
30 min	1.94	0.63	2.02	0.08
90 min	9.18	7.88	7.77	5.82
150 min	43.8	42.50	24.37	22.43
210 min	45.57	44.26	26.14	24.20

**Table 3:** Characteristics of the AEM anion exchange membranes and cation exchange membraneCEMs.

Membrane type Homogeneous	Thickness (μm)	IEC (mmol g^−1^)	Area resistance (Ωcm^2^)	Transport number (%)	pH stability
AEM	125	1.06	1.3	98	2–10
CEM	135	1.04	2.7	97	4–12

**Table 4:** The name of the different factors modification of AEMs.

Named	Factors modification of AEMs			
	Innermost deposition time (h)	Middle electro-deposition time (h)	Middle electro-deposition current (mA/cm^2^)	Outermost deposition time (h)
Modified membrane 1	3	1	5	2
Modified membrane 2	3	1	10	2
Modified membrane 3	3	1	15	2
Modified membrane 4	2	1	15	2
