# Supplementary material for: Mimicking the cell membrane: bio-inspired simultaneous functions with monovalent anion selectivity and antifouling properties of anion exchange membrane
Source: Sci Rep. 2016 Nov 17;6:37285. doi: 10.1038/srep37285 (PMC5112527; doi:10.1038/srep37285)
Supplement: Supplementary Information [file srep37285-s1.pdf]

Scientific Reports

Supporting information

Mimicking the cell membrane: bio-inspired simultaneous functions with monovalent anion selectivity and antifouling properties of anion exchange membrane

Yan Zhao[1], Huimin Liu[1], Kaini Tang[1], Yali Jin[1], Jiefeng Pan[1], Bart Van der Bruggen[2], Jiangnan Shen[1,]* & Congjie Gao[1]

[1]Center for Membrane Separation and Water Science & Technology, Ocean College, Zhejiang University of Technology, Hangzhou 310014, P. R. China

[2]Department of Chemical Engineering, KU Leuven, Celestijnenlaan 200F, B-3001 Leuven, Belgium

*E-mail: shenjn@zjut.edu.cn

1. Polarization current-voltage curves.

0.05 M NaCl/ Na_2SO_4 and 0.5 M Na_2SO_4 were used as test solution and electrode solution respectively. As shown in Fig. S5. The DC with a stepwise increase was supplied by a potentiostat / galvanostat (WY605, EVERFINE PHOTO-E-INFO CO., Ltd.). In this process, the effective area of membrane was 7.065 cm^2 and the voltage beside the AEM was measured by an Avometer. In order to eliminate the possible influence of the temperature, during the experiments the temperature was maintained at 25 ℃. In this experiment, the applied voltage was increased stepwise every 10 s, to allow the membrane reach a steady state. In the whole process, all solution was circulated well by pumps.

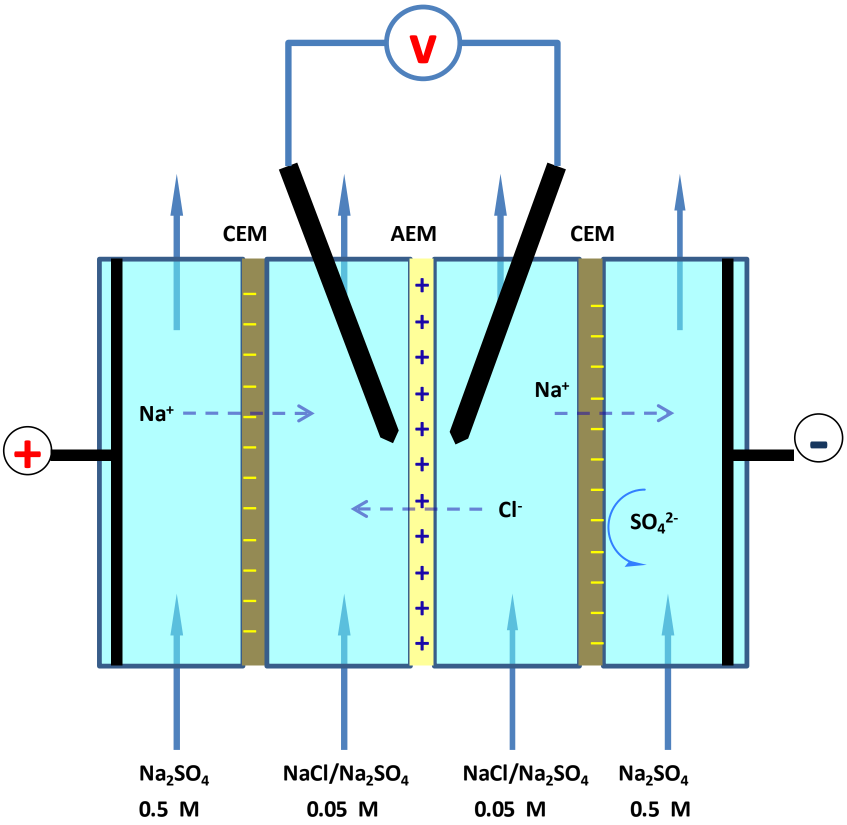

Figure S1. Schematic drawing of a four-electrode mode measurement module for polarization current−voltage curves.

2. Synthesis of N-O-sulfonic acid benzyl chitosan

For the synthesis of NSBC, 2.5 g chitosan was dissolved in 100 mL 0.7 wt% aqueous acetic acid solution, the solution with 1 M HCl solution (pH 3.0). Then 9.675 g 2-formylbenzenesulfonic acid sodium salt was dissolved in 25 mL 60% alcohol solution. After that, the chitosan solution was drop-by-drop added into above solution. Then the mixture solution was refluxed at 60 ℃ for 5 hours. When the solution was cooled to room temperature, 1 M NaOH solution was droply added into it and adjust the pH to 5.0. Then 125 mL $NaBH_4$ solution (2.5% w/v) was added into the solution. Finally, add 250 mL acetone to separate out the white flocculent material. In the end the obtained material was dried in a vacuum oven at 50℃ for 12 hours. The FTIR spectra of NSBC are shown in Fig. S1, the broad band at 3443 cm^{-1} is the stretching vibration of -OH groups in NSBC. The peak at 1200 cm^{-1}, 1036 cm^{-1} and 620 cm^{-1} are actually the symmetric and asymmetric stretching vibration for the $-SO^{3-}$ groups in modified chitosan. Moreover, the characteristic bands for N-H absorption bands at 1640 cm^{-1} indicating the existence of the grafting of sulfonic acid groups onto the chitosan.

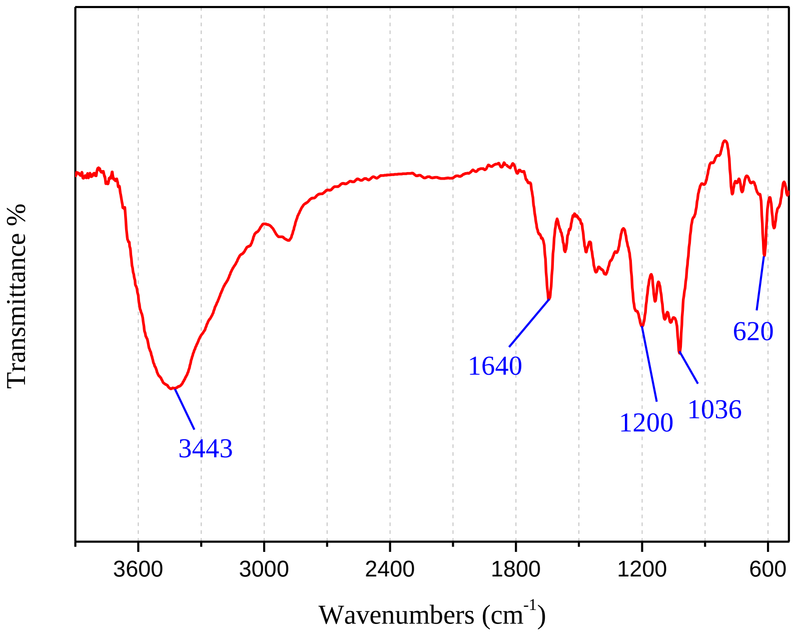

Figure S2. FTIR spectra of NSBC.

3. Permselectivity measurements.

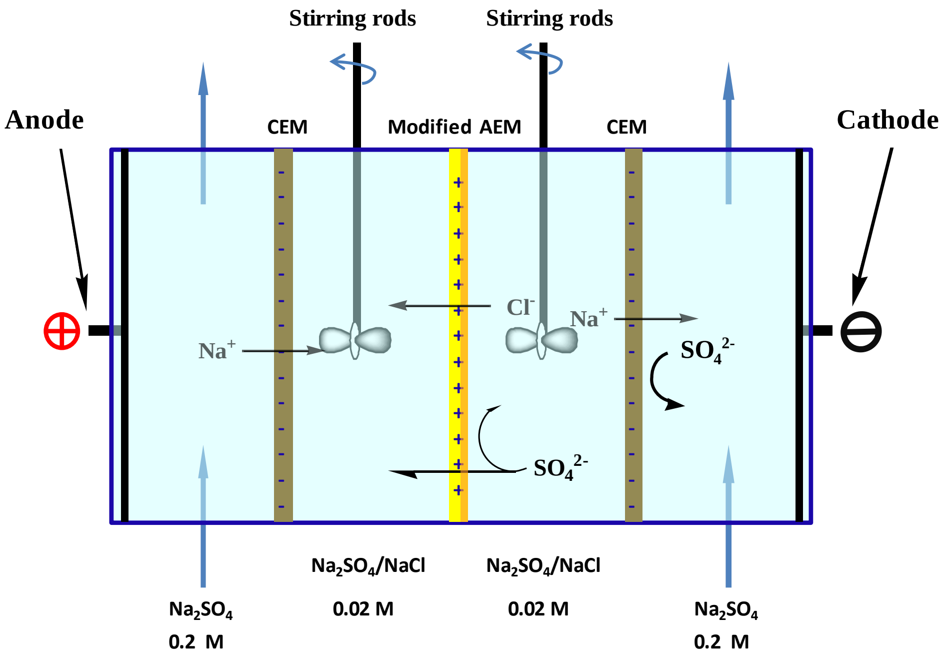

Figure S3. Schematic drawing of a four-electrode mode for monovalent anion permselectivity measurement.

4. Antifouling measurements

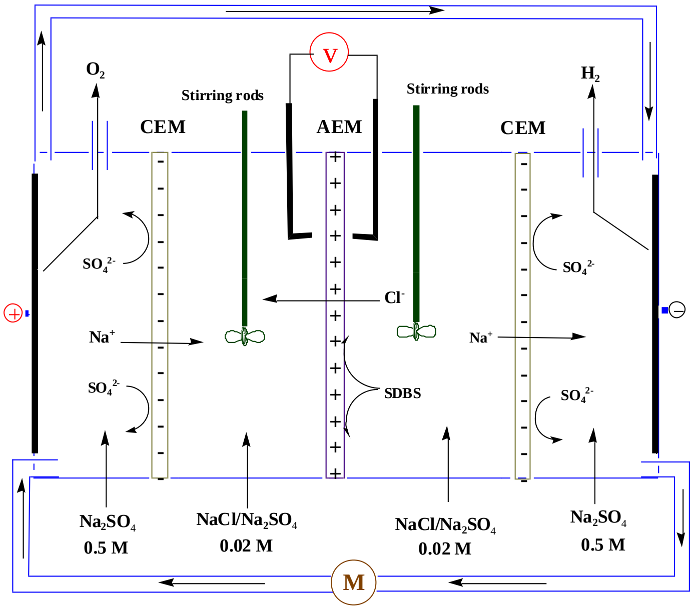

Figure S4. Schematic drawing of a four-compartment experimental setup Antifouling measurements four-compartment experimental setup

5. Custom-designed cell for membrane area resistance measurements

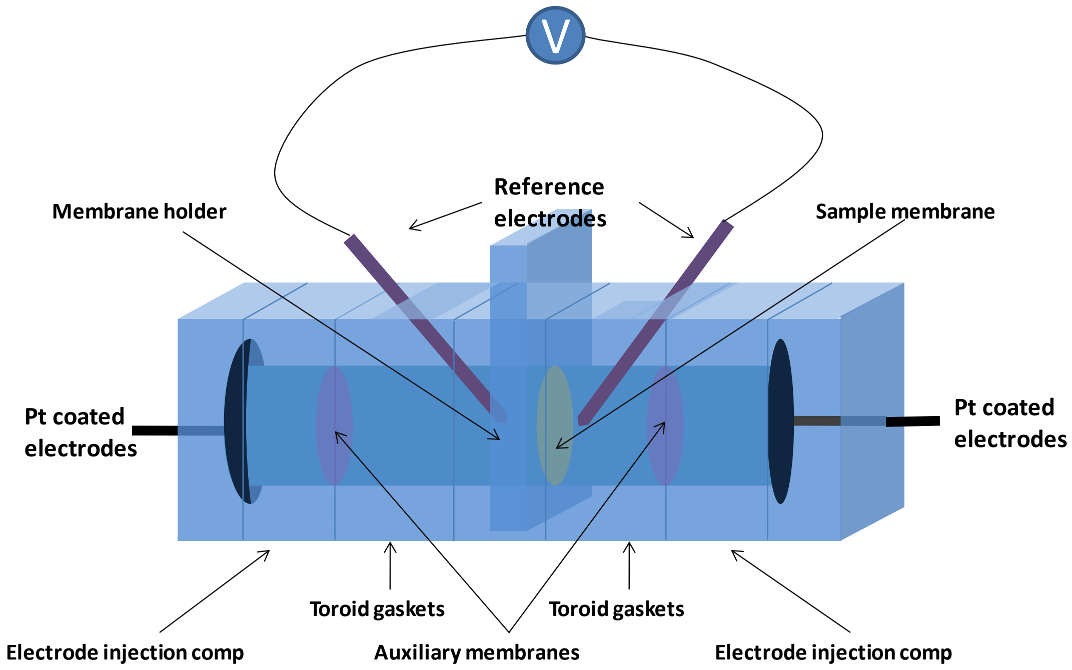

Figure S5. Schematic drawing of custom-designed cell[1].

Reference

1. Hossain, M.M., Wu, L., Li, Y., Ge, L. & Xu, T. Preparation of porous poly(vinylidene fluoride) membranes with acrylate particles for electrodialysis application. *Sep. Purif. Technol.* **150**, 102-111 (2015).
